# Genistein Protects Genioglossus Myoblast Against Hypoxia-induced Injury through PI3K-Akt and ERK MAPK Pathways

**DOI:** 10.1038/s41598-017-03484-4

**Published:** 2017-07-11

**Authors:** Wanghui Ding, Xiaoyan Chen, Wen Li, Zhen Fu, Jiejun Shi

**Affiliations:** Department of Orthodontics, School of Stomatology affiliated to Medical College, Zhejiang University, Hangzhou City, Zhejiang Province China

## Abstract

Obstructive sleep apnea and hypopnea syndrome (OSAHS) is a clinical syndrome characterized by recurrent episodes of obstruction of the upper airway during sleep that leads to a hypoxic condition. Genioglossus, an important pharyngeal muscle, plays an important role in maintaining an open upper airway for effective breathing. Our previous study found that genistein (a kind of phytoestrogen) protects genioglossus muscle from hypoxia-induced oxidative injury. However, the underlying mechanism is still unknown. In the present study, we examined the effects of hypoxia on genioglossus myoblast proliferation, viability and apoptosis, and the protective effect of genistein and its relationship with the PI3K/Akt and ERK MAPK pathways. Cell viability and Bcl-2 were reduced under hypoxic condition, while ROS generation, caspase-3, MDA, and DNA damage were increased following a hypoxia exposure. However, the effects of hypoxia were partially reversed by genistein in an Akt**-** and ERK**-** (but not estrogen receptor) dependent manner. In conclusion, genistein protects genioglossus myoblasts against hypoxia-induced oxidative injury and apoptosis independent of estrogen receptor. The PI3K-Akt and ERK1/2 MAPK signaling pathways are involved in the antioxidant and anti-apoptosis effect of genistein on genioglossus myoblasts.

## Introduction

Obstructive sleep apnea (OSAS) is a result of repeated episodes of upper airway (UA) obstruction during sleep, and it is considered to be a risk factor for cardiovascular disorders with life-threatening consequences^1^. OSAS is categorized as a hypoxic condition^1, 2^, which can impair UA muscle function^4^. UA dilator muscles of individuals with OSAS demonstrate an increased level of activity during wakefulness, which induces muscle fatigue^5, 6^. Furthermore, this hyperactivity not only leads to changes in contractile protein composition, but also muscle fiber damage^7, 8^.

Genioglossus is an important pharyngeal muscle that plays a crucial role in maintaining an open UA for effective breathing^9, 10^. Genioglossus muscle fatigue is increased in individuals with OSAS, which increases the likelihood of UA collapse especially during frequent and prolonged apnea, leading to a vicious cycle of further airway obstruction and muscle dysfunction^4, 11^. Genioglossus is under a hypoxia environment in OSAS patients, and degenerative morphological changes of genioglossus takes place such as myofibril discontinuities, dilation of mitochondria and disruption of cristae that parallel the changes of muscle fatigue^12^. In addition, oxidative stress and decreased cellular antioxidant capacity have been demonstrated in patients with OSAS^13–15^. Therefore, it is hypothesized that genioglossus muscle dysfunction is associated with a hypoxia-induced oxidative injury.

Genistein, a soy isoflavone, is a plant-derived polyphenolic non-steroidal compound with estrogen-like biological activity without side effect for long-term use^16, 17^. We previously found that genistein attenuates genioglossus muscle fatigue *in vivo* ^4, 12^. However, the detailed molecular mechanisms that underlie the effects of genistein on genioglossus remain to be elucidated. Furthermore, the PI3K/Akt and MAPK pathways regulate a variety of cellular activities including proliferation, differentiation, survival, oxidative stress, and death^18–20^. Genistein has been found to exert its protective effects in relation to the PI3K/Akt and MAPK pathways^21–24^. Therefore, the purpose of this study is to determine the effects of hypoxia on genioglossus myoblast viability, proliferation and apoptosis, and to identify the muscle protective effect of genistein and its underlying relationship with the PI3K/Akt and MAPK pathways.

## Materials and Methods

### Materials

Sprague–Dawley (SD) rats were obtained from the Experimental Animal Center of Second Military Medical University (Shanghai, China). All animal care and use were conducted according to the National Research Council guidelines and approved by the Animal Care and Use Committee of Zhejiang University. The cell culture medium, class II collagenase, trypsin and fetal bovine serum (FBS) were from Life Technologies (Grand Island, NY, USA). The 2′,7′-dichlorofluorescein diacetate (DCF-DA) was obtained from Molecular Probes (Eugene, Oregon, USA). The genistein, ICI 182780, SB 203580, and SP 600125 were obtained from Sigma Chemical Company (St Louis, MO, USA). The wortmannin, U0126, and antibodies of phospho-Akt, phospho-ERK1/2, and Bcl-2 were obtained from Cell Signaling Technology (Danvers, MA, USA). The anti-rabbit I horseradish peroxidase (HRP)-conjugated goat antibodies were obtained from Santa Cruz Biotechnologies (Heidelberg, Germany). The MTT, DAPI, MDA and caspase-3 detecting kit were obtained from Beyotime Institute of Biotechnology (Jiangsu, China). The BrdU detection kit was from obtained Boehringer Mannheim Biochemica (Mannheim, Germany).

### Primary culture of genioglossus myoblast and hypoxia exposure

The genioglossus muscle tissues were obtained from newborn (3–5 d) SD rats. They were trimmed of excessive connective and fat tissues, hand-minced with a sterile scissor into approximately 1 mm^3^, and washed with PBS for three times. Enzymatic dissociation was performed by two-step digestion of minced muscles at 37 °C in 0.5% collagenase type II for 20 min and 0.05% trypsin for 10 min. The digestion was terminated by growing medium (GM, high glucose DMEM containing 10% FBS and 1% penicillin-streptomycin), and cells were separated from muscle fiber fragments and tissue debris by 120 mesh screen cloth. The cells were centrifuged, and plated in tissue culture dishes coated with poly-lysine in GM. Cells were incubated in normoxic condition with 37 °C and 5% CO_2_. However, hypoxic conditions were created in the hypoxia chamber with 0.5% O_2_ and 5% CO_2_.

### Detection of cell proliferation

The cells were seeded into 96-well cell culture plates at a density of 2 × 10^4^ cells/well. After 24 h culture at 37 °C and 5% CO_2_, 10 μM of genistein (the concentration was decided by our previous study) were added to the plates_._ After pretreated with genistien for 1 h, the cells were incubated under normoxic or hypoxic conditions for another 24 h, 48 h, and 72 h. MTT assay was performed following the well-described procedure^25^. Briefly, the wells were washed three times with DMEM. Then, 180 μl aliquots of DMEM and 20 μl aliquots of MTT solution (5 mg/ml of PBS) were added to each well at the established time. After 4 h of incubation at 37 °C and 5% CO_2_, the media were removed and formazan crystals were solubilized with 150 μl DMSO. The plates were read on ELx800 absorbance microplate reader (Bio-Tec Instruments INC, USA) at 550 nm wavelength. The results of MTT assay were verified by BrdU DNA incorporation assay via detection of DNA synthesis^26^. The incorporated BrdU was measured using a BrdU detection kit according to the manufacturer’s instructions. The absorbance of the extract was read at a wavelength of 450 nm with a reference wavelength of 630 nm.

### DAPI staining

Cells were seeded onto 6-well tissue culture plates at a concentration of 1 × 10^5^ cells/well and incubated under normoxia or hypoxia conditions at the presence or absence of genistein for 48 h. At the end of incubation, cells were fixed with ice-cold 4% paraformaldehyde for 20 min, permeabilized with 0.1% Triton x-100 for 25 min and washed with ice-cold PBS three times. At last the cells were stained with 4′, 6-diamidino-2-phenylindole (DAPI, 5 μg/ml) for 25 min and observed under a fluorescence microscope with a peak excitation wave length of 340 nm. Nuclei were identified as normal, fragmented, or condensed. Fragmented or condensed nuclei were classified as apoptotic. The results were expressed as a percentage of apoptotic cells. A minimum of 500 cells were counted for each treatment from at least three independent experiments.

### Flow cytometry analysis

Cells were seeded onto 6-well tissue culture plates at a concentration of 1 × 10^5^ cells/well and incubated under normoxia or hypoxia conditions at the presence or absence of genistein for 48 h. At the end of incubation, trypsin digestion was performed to detach the cells from culture plates. We resuspended the cells in 1x binding buffer and adjusted the concentration to 1 × 10^6^ cells/mL. Cell suspensions were added to 5 mL Falcon tubes containing 5 μL of FITC-Annexin V and 5 μL of PI dye and then gently mixed. Following incubation in the dark at room temperature (~25 °C) for 15 min, 400 μL of 1x binding buffer was added to each tube, and the samples were analyzed within 1 h using a flow cytometer (BD Biosciences).

### Measurements of ROS generation

Changes in intracellular ROS levels were determined by measuring the oxidative conversion of cell permeable 2′,7′-dichlorofluorescein diacetate (DCF-DA) to fluorescent dichlorofluorescein (DCF). Cells in 12 well culture dishes were incubated at normoxia or hypoxia conditions at the presence or absence of genistein for 24 h, 48 h, and 72 h. The cells were washed with PBS and incubated with DCF-DA at 37 °C for 20 min. Then, DCF fluorescence distribution of 20,000 cells was detected by a fluorescence microplate reader at an excitation and emission wavelength of 488 nm and 525 nm, respectively.

### Determination of MDA production

Concentration of malondialdehyde (MDA), an index of lipid peroxidation, was determined spectrophotometrically according to the Draper and Hadley method^27^. A 0.1 mL aliquot of genioglossus myoblast extract supernatant was mixed with 0.2 mL of 0.37% trichloroacetic acid (TBA) solution, and the mixture were incubated for 10 min at 100 °C and cooled. Then, the mixture was centrifuged at 1,000 g for 10 min, and 0.2 mL aliquot of the mixture was added to 96 well. Absorbance of TBA-MDA complex was determined at 532 nm using a microplate reader.

### Caspase fluorometric assay

Measurement of caspase-3 activity was performed as previously described^28^. Briefly, cytosolic extract (100 μg protein) was incubated for 1 h at 37 °C with the reaction buffer (25 mM HEPES, pH 7.5, 10% sucrose, 0.1% CHAPS, 5 mM DTT and 5 mM EDTA) in a total volume of 150 μl containing 25 μM acetyl-Asp-Glu-Val-Asp-p-nitroanilide (Ac-DEVD-pNA). Enzyme-catalyzed release of p-nitroanilide was measured at 405 nm by a microplate reader.

### Western blot analysis

Cells were harvested into a lysis buffer consisting of 2.5 mM HEPES, pH 7.5, 10% glycerol, 5 mM EDTA, 5 mM EGTA, 100 mM NaCl, 100 mM Na pyrophosphate, 50 mM NaF, 0.1 mM NaVO4, 1% Triton X-100, 1 mM benzamidine, 1 mM phenylmethylsulfonyl fluoride, 10 μg/ml leupeptin, 10 μg/ml aprotinin, and 2 μg/ml pepstatin. Equal protein amounts (50 μg) of genioglossus homogenates were electrophoresed through 12% SDS-polyacrylamide gel and electroblotted onto polyvinylidene fluoride (PVDF) membranes (Millipore, USA). The blots were then washed in PBST (PBS with 0.05% Tween-20), blocked with 5% fat-free milk in PBST for 1 h, and incubated overnight with an appropriate primary antibody at the dilutions recommended by the supplier (1:1000). The detection was made with HRP-conjugated secondary antibodies (1:10000) and ECL System by Kodak IMAGE STATION 2000 MM (Kodak, Rochester, NY, USA).

### Statistical analysis

Statistical analyses were performed using the SPSS Statistical Analysis Software, version 13.0. All data are expressed as the means ± the standard deviation from three independent experiments. Statistical significance of differences was calculated using ANOVA followed by Tukey’s HSD post hoc test. Statistical significance was established at P < 0.05.

## Results

### Oxidative stress and genioglossus myoblast injury can be induced by Hypoxia

**Figure 1:**
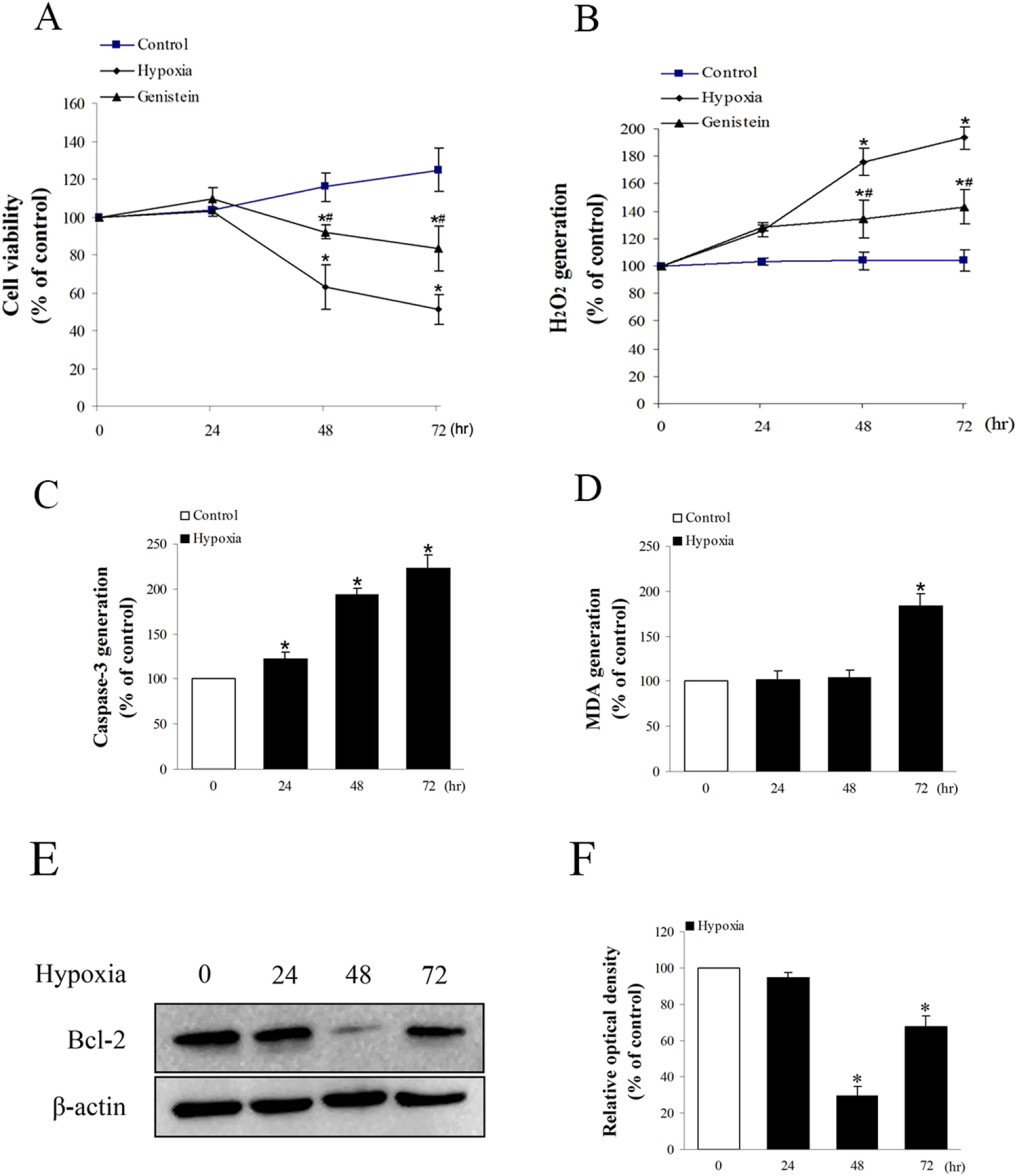
The time-dependent effects of hypoxia exposure on cell viability and cell injury. (**A**) The effects of hypoxia on cell viability. Cell viability was significantly reduced by 48 h and 72 h of hypoxia. (**B**) The effects of hypoxia on H_2_O_2_ generation. H_2_O_2_ generation was increased by hypoxia in a time-dependent manner. (**C**) The effects of hypoxia on caspase-3 activity. Caspase-3 activity was increased by hypoxia in a time-dependent manner. (**D**) The effects of hypoxia on MDA generation. MDA was unchanged after 24 h and 48 h hypoxia exposure, while it was significantly increased at 72 h. (**E**,**F**) The effects of hypoxia on the Bcl-2 protein expression. The level of Bcl-2 was reduced at 48 h, and it was partially increased at 72 h. *P < 0.05 versus control group; ^#^P < 0.05 versus hypoxia group.

Genioglossus myoblast were incubated in hypoxic conditions of 0.5% O_2_ and 5% CO_2_ for 24 h, 48 h, and 72 h. Cell viability unchanged following 24 h hypoxic exposure, while it was significantly decreased after 48 and 72 h (Fig. 1A). The levels of H_2_O_2_ was measured using DCF-DA to determine whether hypoxia stimulates the generation of ROS in genioglossus myoblast. The data showed that the intracellular H_2_O_2_ was significantly increased in a time-dependent manner (0–72 h) (Fig. 1B). Caspase 3 plays an important role in apoptosis and is responsible for chromatin condensation and DNA fragmentation^29^. Our data showed that the level of caspase 3 expression was increased as the extension of the time under the hypoxic conditions (0–72 h) (Fig. 1C). Besides, the index of lipid peroxidation, MDA, was increased following 72 h hypoxia exposure (Fig. 1D). B-cell lymphoma protein-2 (Bcl-2), as a regulator of permeabilization of the mitochondrial outer membrane and the consequent release of cytochrome c, prevents apoptosis and hypoxia-induced injury^30, 31^. The level of Bcl-2 was reduced at 48 h, but it was increased at 72 h. However, the level of Bcl-2 remained less than normal value (Fig. 1E,F).

### Effects of genistein on hypoxia-induced apoptosis of genioglossus myoblast

**Figure 2:**
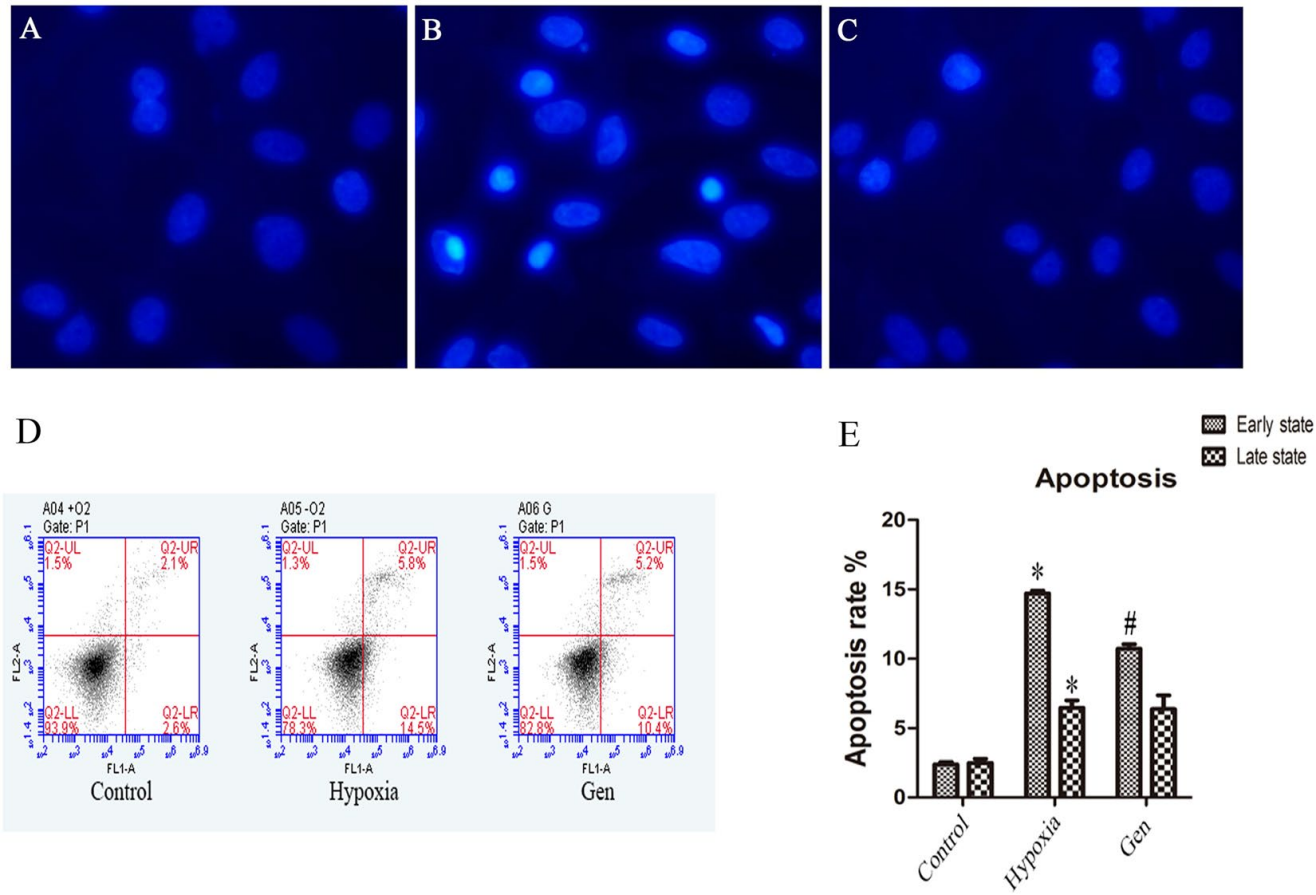
The effects of genistein on hypoxia-induced apoptosis. (**A**,**B**) Nuclei have the normal phenotype demonstrating bright colors and homogeneity at normoxic and 24 h hypoxic conditions. Nuclei that emitted bright fluorescence and condensed were classified as apoptotic. The apoptotic nuclei were increased after 48 h hypoxia conditions. (**C**) The number of apoptotic nuclei was reduced by genistein treatment. (**D**,**E**) The effects of genistein on hypoxia-induced apoptosis by Annexin V-FITC/PI flow cytometry analyses. Early-stage and late-stage apoptosis rates were significantly increased after 48 h hypoxia exposure. The apoptotic nuclei were increased after 48 h hypoxia exposure. Genistein significantly decreased the early-stage apoptosis of genioglossus myoblasts. *P < 0.05 versus control group; ^#^P < 0.05 versus hypoxia group.

DAPI staining and Annexin V-FITC/PI flow cytometry analysis were used to detect the effects of hypoxia on the nuclear changes and apoptosis of genioglossus myoblast. As shown in Fig. 2A and B, nuclei had the normal phenotype demonstrating bright and homogenously at normoxic or 24 h hypoxic condition. But we found that this process was inhibited by genistein (Fig. 2C). Nuclei that emitted bright fluorescence, fragmented, or condensed were classified as apoptotic. The results from flow cytometry analyses indicated that the early-stage and late-stage apoptosis rate were significantly increased after 48 h hypoxia exposure. Genistein significantly decreased the early-stage apoptosis of genioglossus myoblasts (Fig. 2D,E).

### Relationship between hypoxia-induced cell injury, genistein and estrogen receptor (ER)

**Figure 3:**
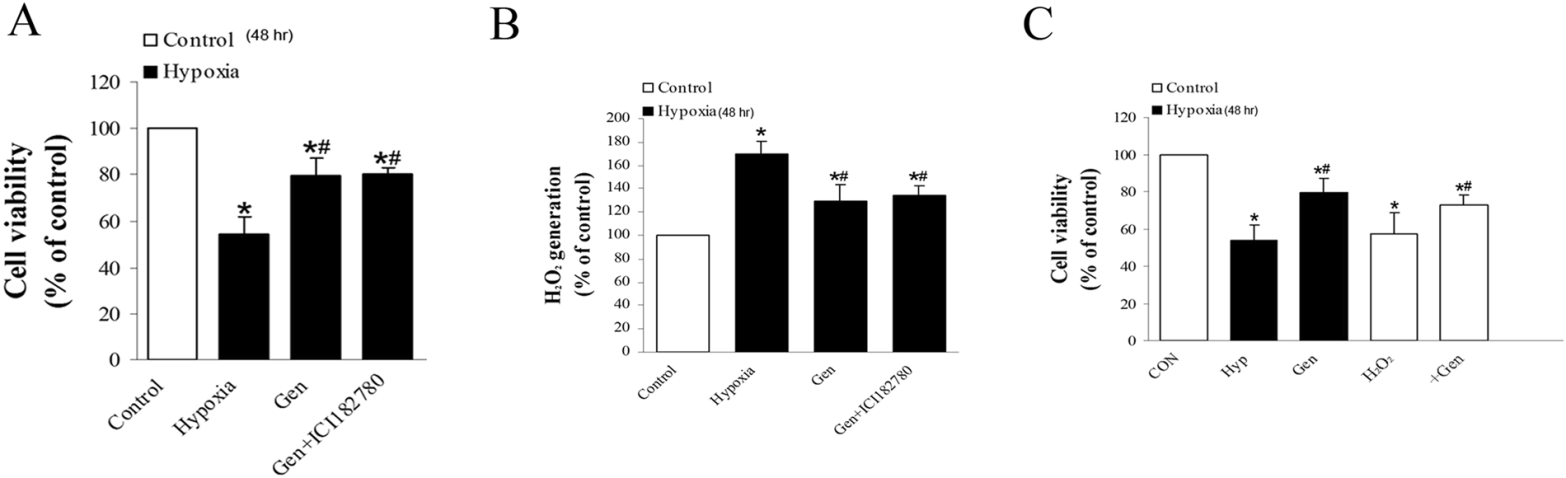
The relationship between hypoxia-induced cell injury, genistein and estrogen receptor (ER). (**A**) The effects of genistein on cell viability. Cell viability was significantly reduced by hypoxia. Genistein partially reversed the hypoxia-induced cell injury independent of ER. (**B**) The effects of genistein on hypoxia-induced H_2_O_2_ generation. Genistein reduced the hypoxia-induced H_2_O_2_ generation at 48 h independent of ER. (**C**) Exogenous H_2_O_2_ (100 μM) was used as a positive control for assessing the effect of hypoxia-induced H_2_O_2_ on cell viability. *P < 0.05 versus control group; ^#^P < 0.05 versus hypoxia group.

In order to observe the relationship between ER and genistein on hypoxia-induced genioglossus myoblast, cells were treated with genistein and ICI 182780 (ER antagonist, 100 nM). The inhibition of hypoxia on cell viability was attenuated after cells were treated by genistein with or without ICI 182780. Results showed genistein attenuated hypoxia-induced H_2_O_2_ generation and its effect on cell viability was not influenced by ICI 182780 (Fig. 3A,B). Exogenous H_2_O_2_ (100 μM) was used as a positive control for assessing the effect of hypoxia-induced H_2_O_2_ generation on the cell viability (Fig. 3C).

### Relationship among the ERK1/2-MAPK and PI3K-Akt pathways, cell injury, and apoptosis

**Figure 4:**
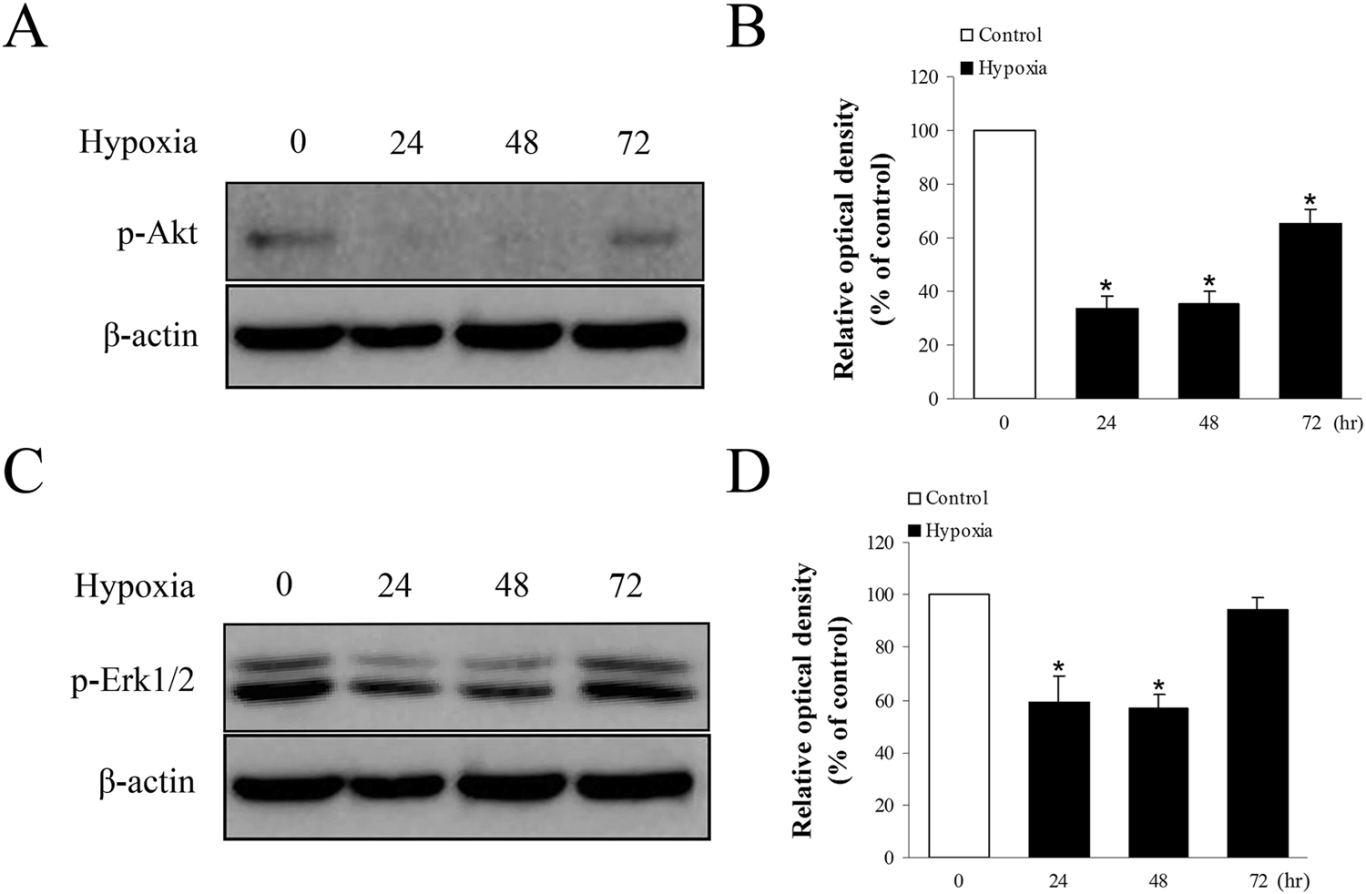
The effects of hypoxia on p-Akt and p-ERK1/2 proteins. (**A**,**B**) The effects of hypoxia on p-Akt protein. P-Akt was remarkably reduced after 24 h and 48 h hypoxia exposure, while it was considerately recovered at 72 h. (**C**,**D**) The effects of hypoxia on p-ERK1/2 protein. P-ERK1/2 was significantly reduced after 24 h and 48 h hypoxia conditions, while it was returned to normal at 72 h. *P < 0.05 versus control group; ^#^P < 0.05 versus hypoxia group.

The signaling molecules associated with hypoxia-induced cell injury and apoptosis, p38, ERK1/2, JNK MAPK, and PI3K-Akt were evaluated. No changes were detected in p38 and JNK MAPK proteins under hypoxic conditions (data not shown). Meanwhile, we found phospho-Akt and phospho-ERK1/2 were remarkably reduced after 24 h and 48 h of hypoxia, but they were recovered at 72 h (Fig. 4A–D).

**Figure 5:**
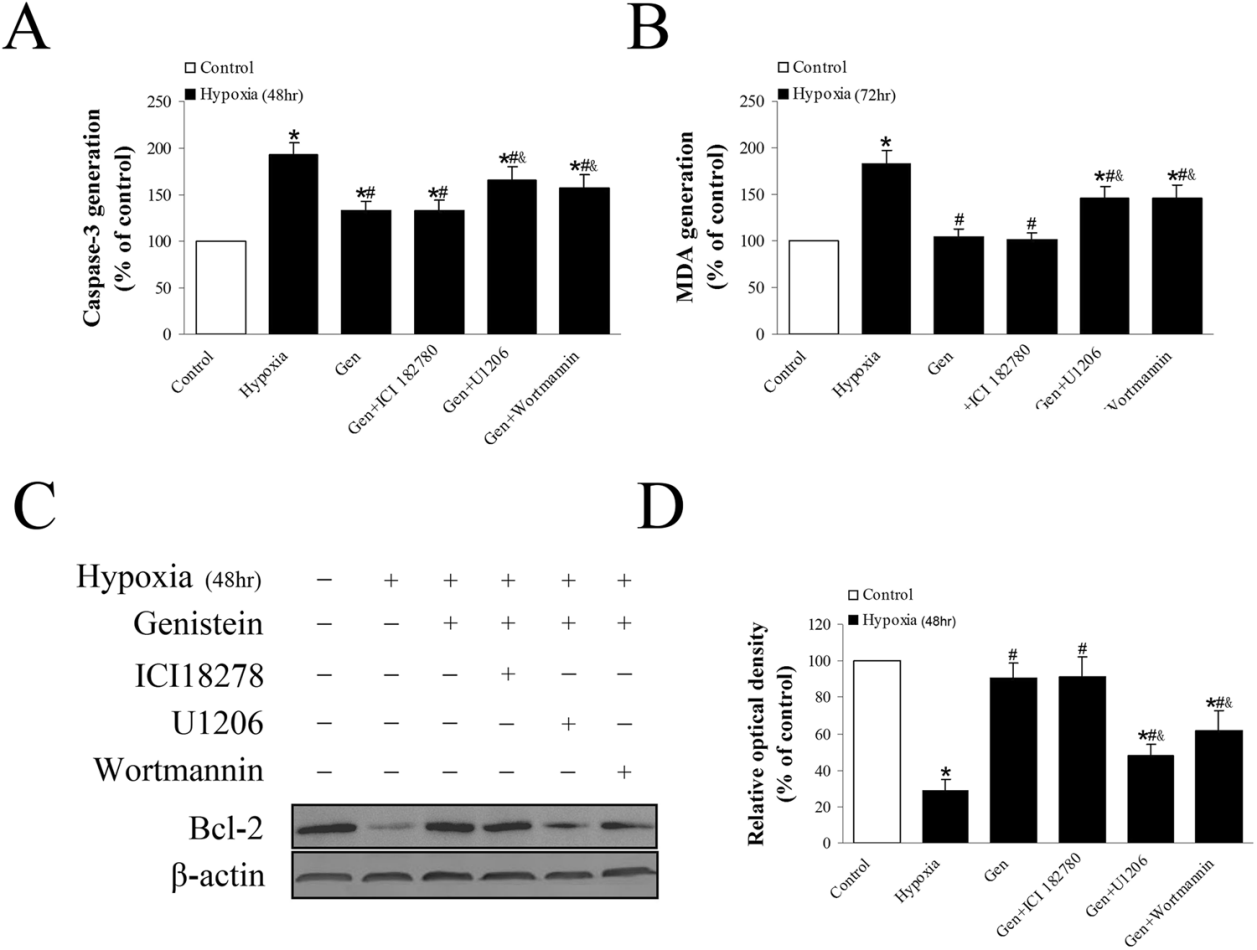
The relationship between apoptosis-related proteins, genistein and ER, PI3K-Akt, and ERK1/2 MAPK pathways. (**A**) The inhibitory effect of genistein on caspase-3 activity was partially inhibited by wortmannin and U0126. This effect was unaffected by ICI 182780. (**B**) The inhibitory effect of genistein on MDA generation was partially reversed by wortmannin and U0126. This effect was unaffected by ICI 182780. (**C**,**D**) Genistein increased the expression of Bcl-2 protein under hypoxic condition. However, this effect was reversed by wortmannin and U0126 but unaffected by ICI 182780. *P < 0.05 versus control group; ^#^P < 0.05 versus hypoxia group; ^&^P < 0.05 versus Gen group.

**Figure 6:**
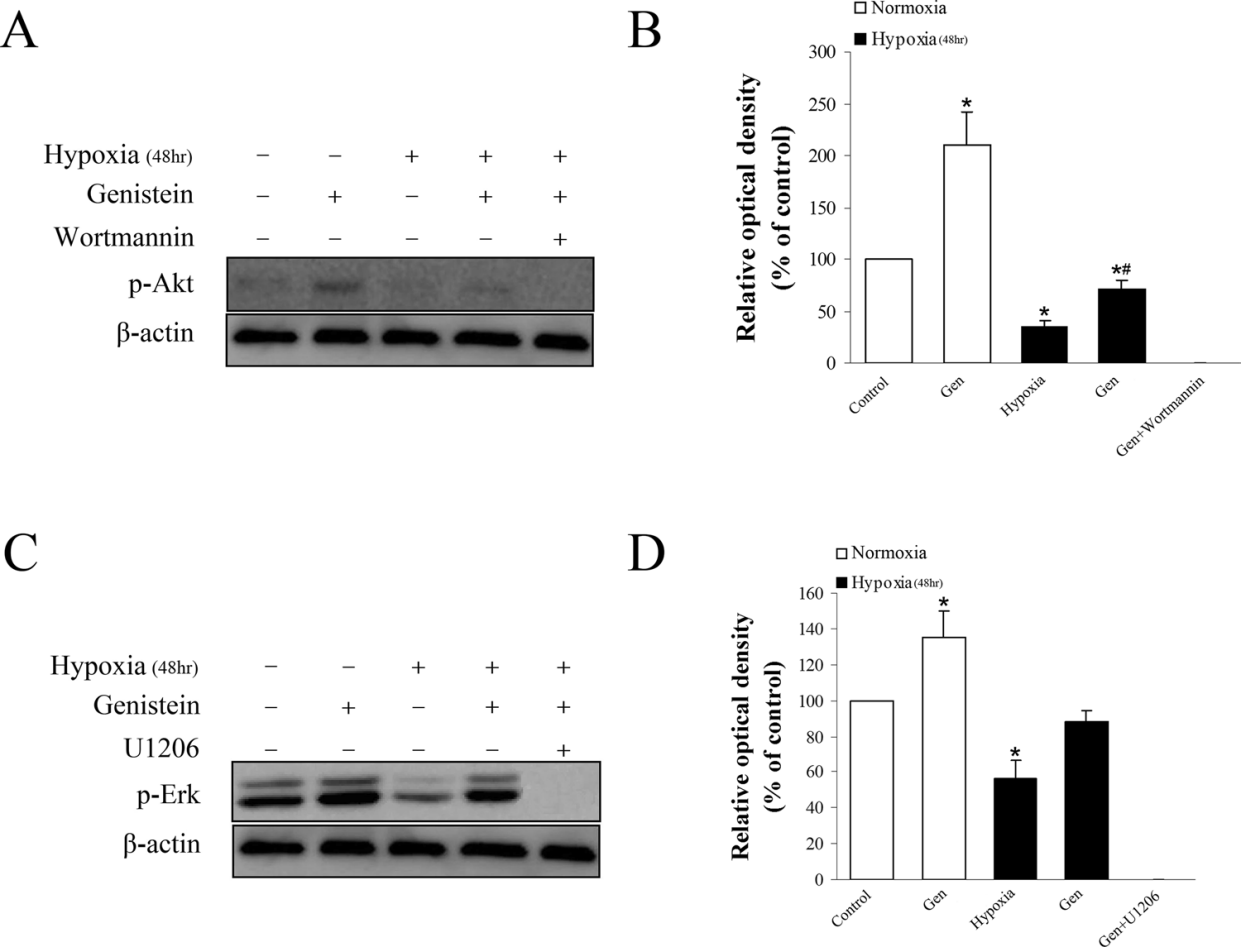
The effects of genistein on the PI3K-Akt and ERK1/2 MAPK pathways under normoxic or hypoxic condition. (**A**,**B**) P-Akt was remarkably increased by genistein under 48 h normoxic condition. It was partially reversed by genistein under 48 h hypoxic condition. Wortmannin completely inhibited the expression of p-Akt (**C**,**D**) P-ERK1/2 was increased by genistein under 48 h normoxic condition. It was reversed by genistein under 48 h hypoxic condition. U1206 completely inhibited the expression of p-ERK1/2. *P < 0.05 versus control group; ^#^P < 0.05 versus hypoxia group.

To further validate the interaction between genistein and pathways, we treated cells with or without genistein, ICI 182780, wortmannin (1 μM, Akt inhibitor), U0126 (1 μM, MEK inhibitor which inhibits ERK1/2). Hypoxia exposure induced apoptosis of genioglossus myoblasts through up-regulation of caspase 3 and down-regulation of Bcl-2. Genistein treatment reduced the expression of caspase-3 and MDA, while wortmannin and U0126 increased it (Fig. 5A,B). The expression of Bcl-2 was increased by genistein, unaffected by ICI 182780, but reduced by wortmannin and U0126 (Fig. 5C,D). U0126 and wortmannin partially reversed the effects of genistein on hypoxia-induced injury through inhibiting ERK and Akt phosphorylation (Fig. 6A–D). In summary, genistein exerted an anti-apoptotic effect on genioglossus myoblasts under hypoxic conditions by activating PI3K-Akt and ERK1/2-MAPK pathway, but independent of ER.

## Discussion

OSAS is associated with oxidative stress and UA muscle fatigue^12^. Furthermore, it is reported that oxidative-stress contributes to hypoxia-induced respiratory muscle impairment in an animal model of OSAS^32^. However, the underlying mechanism is still unknown. Genioglossus is a very important UA dilating muscle, which protrudes the tongue, increases the oropharyngeal size, and decreases airway collapsibility. Therefore, genioglossus plays a significant role in maintaining an open UA for effective breathing^9^. We established a genioglossus myoblast culture model under hypoxic conditions to detect the underlying mechanism of oxidative stress and muscle injury. The results of this study demonstrate that genistein partially protects genioglossus myoblast against hypoxia-induced oxidative stress injury. This protective effect is mediated by the regulation of ROS, lipid peroxidation, Bcl-2, and caspase-3 through the PI3K-Akt and ERK1/2 MAPK signaling pathways, which suggests that genistein is effective against oxidative stress through multiple ways in genioglossus myoblast.

This study found that hypoxic exposure stimulates the generation of ROS, which is coincident with OSAS and the animal model of the disorder^33, 34^. ROS includes superoxide anion, hydrogen peroxide and hydroxyl radical, and plays a physiological role in the function of a number of cellular signaling pathways^35^. However, excessive ROS induces cell injury including DNA damage, lipid peroxidation, and apoptosis. In this study, it was detected that hypoxic exposure significantly reduces the viability of genioglossus myoblasts. Apoptotic nuclei were increased by hypoxic exposure, indicating that hypoxia induces apoptosis of genioglossus myoblasts. In addition, caspase-3, an apoptosis marker, was also increased under hypoxic conditions. Bcl-2, an anti-apoptosis protein, was reduced under a hypoxic condition though it recovered partially with a longer exposure. MDA, the index of lipid peroxidation, was increased after 72 h of hypoxia. Therefore, we conclude that hypoxia induces oxidative cell injury in a ROS-dependent manner, leading to apoptosis through down-regulation of Bcl-2 and up-regulation of caspase-3, consistent with previous studies^36^.

Genistein, an isoflavone phytoestrogen, is a biologically active plant substance with a chemical structure similar to that of endogenous estrogen. In this study, we found that genistein attenuates the deleterious effects of hypoxia on cell viability, which is ROS-dependent but not ER-dependent. This discovery is different from the finding of a previous study which determined that genistien exerts protective effect on oxidative stress-induced human endothelial cells (HUVECs) injury through ERs^37^. The reason might be that genistein has a relatively high binding affinity for ER β. ER β is highly expressed in HUVECs, but lowly expressed in skeletal muscles^38–40^. In addition, genistein increased Bcl-2 levels and attenuated caspase-3 activity and lipid peroxidation independent of ERs. This suggests that anti-apoptotic and antioxidant actions represent two of the most important mechanisms by which genistein protect genioglossus myoblasts against a hypoxic injury. The antioxidant property of genistein has been demonstrated in several studies and is reported to be more effective than that of antioxidant vitamins and estrodiol in scavenging ROS and lipid peroxidation^41–43^. However, other research indicates that genistein stimulates the expression of apoptotic markers such as caspase-3. Taken together, these observations implicate that variation in the dosage of genistein and cell type used in experiments can result in a totally distinct outcome. The antioxidant property probably accounts for the protective effect of genistein on genioglossus fatigue which was found in a previous study^44^. Furthermore, the result is consistent with the finding that superoxide scavengers attenuate rat pharyngeal dilator muscle fatigue in hypoxia^45^.

It has been determined that the MAPK and PI3K/Akt signaling pathways regulate a variety of cellular activities including proliferation, differentiation, survival, and death^46, 47^. The pathways play a central role in orchestrating growth, proliferation, and anti-apoptotic mechanisms to promote cell cycle and survival. These cellular processes are activated by diverse extracellular and intracellular stimuli including peptide growth factors, cytokines, hormones, and various cellular stressors such as oxidative stress and endoplasmic reticulum stress. The mammalian family of MAPK includes extracellular signal-regulated kinase (ERK1/2), p38, and c-Jun NH_2_-terminal kinase (JNK). In this study, we found that p38 and JNK MAPK were not involved in hypoxia-induced genioglossus myoblast injury. This finding is different from previous research showing that p38 and JNK are involved in hypoxia-induced hepatocyte injury, which suggests that the signaling pathways related to hypoxia are cell-specific^36^. Hypoxia decreased the expression of the phospho-Akt and phospho-ERK1/2, and Akt and ERK inhibitor (wortmannin and U0126) partially reversed the protection of Gen on the hypoxia-induced injury. This result suggests that the PI3K/Akt and ERK MAPK pathways are involved in the hypoxia injury and protective effect of genistein on genioglossus myoblasts. The conclusion can be verified by the result that genistein increased the expression of phospho-Akt and phospho-ERK1/2, and that wortmannin and U0126 reversed the effect of genistein on Bcl-2, caspase-3, and MDA. Therefore, the effects of genistein on hypoxia-induced genioglossus myoblast injury are presumably through non-genomic pathways rather than ER-mediated genomic pathways. Signaling pathways including the ERK1/2 and PI3K/Akt cascades permit non-genomic actions of genistein.

In conclusion, genistein protects primary cultured genioglossus myoblasts against hypoxia-induced oxidative injury and cell apoptosis independent of ER. The PI3K-Akt and ERK1/2-MAPK signaling pathways are involved in the antioxidant and anti-apoptotic effects of genistein on genioglossus myoblasts under hypoxic conditions.
